# Supplementary material for: BMP signaling promotes zebrafish heart regeneration via alleviation of replication stress
Source: Nat Commun. 2025 Feb 17;16:1708. doi: 10.1038/s41467-025-56993-6 (PMC11832743; doi:10.1038/s41467-025-56993-6)
Supplement: Supplementary file 1 — Supplementary Information [file 41467_2025_56993_MOESM1_ESM.pdf]

Supplement

Table of contents

Supplementary Figure 1	page 2
Supplementary Figure 2	page 3
Supplementary Figure 3	page 4
Supplementary Figure 4	page 5
Supplementary Figure 5	page 6
Supplementary Figure 6	page 8
Supplementary Figure 7	page 9
Supplementary Figure 8	page 10
Supplementary Figure 9	page 11
Supplementary Figure 10	page 12
Supplementary References	page 13
Supplementary Table 1	page 14
Supplementary Table 2	page 14
Supplementary Table 3	page 15
Supplementary Table 4	page 16

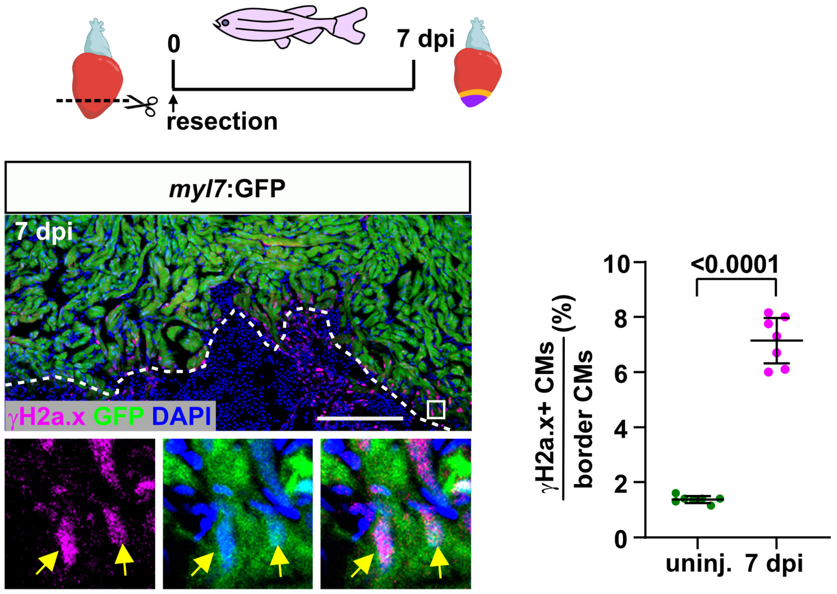

Supplementary Figure 1. *γH2a.x accumulates in wound border cardiomyocytes in hearts that regenerate after ventricular resection*

Immunofluorescence on cryosections of *myl7*:GFP transgenic hearts reveals γH2a.x accumulation (arrows) in GFP+ CMs at the wound border at 7 days post ventricular resection (dpi). White box in the representative image indicates magnified region. Plot shows fraction of γH2a.x+ CMs out of all CMs within 150 µm of the wound border. Data points represent fraction in percent derived from individual hearts. Data are presented as mean values. Error bars, confidence interval (CI) 95%; Student´s t-test. n_E = 1, n_A = 7 uninjured, 7 resection, n_C (analyzed CMs) = 5810 uninjured, 6120 resection. Scale bar, 150 µm. Source data are provided in the Source Data file. Some elements were created in BioRender (Agreement number: AH27UPOG7H; Posadas, D. (2025) https://BioRender.com/w88l548)

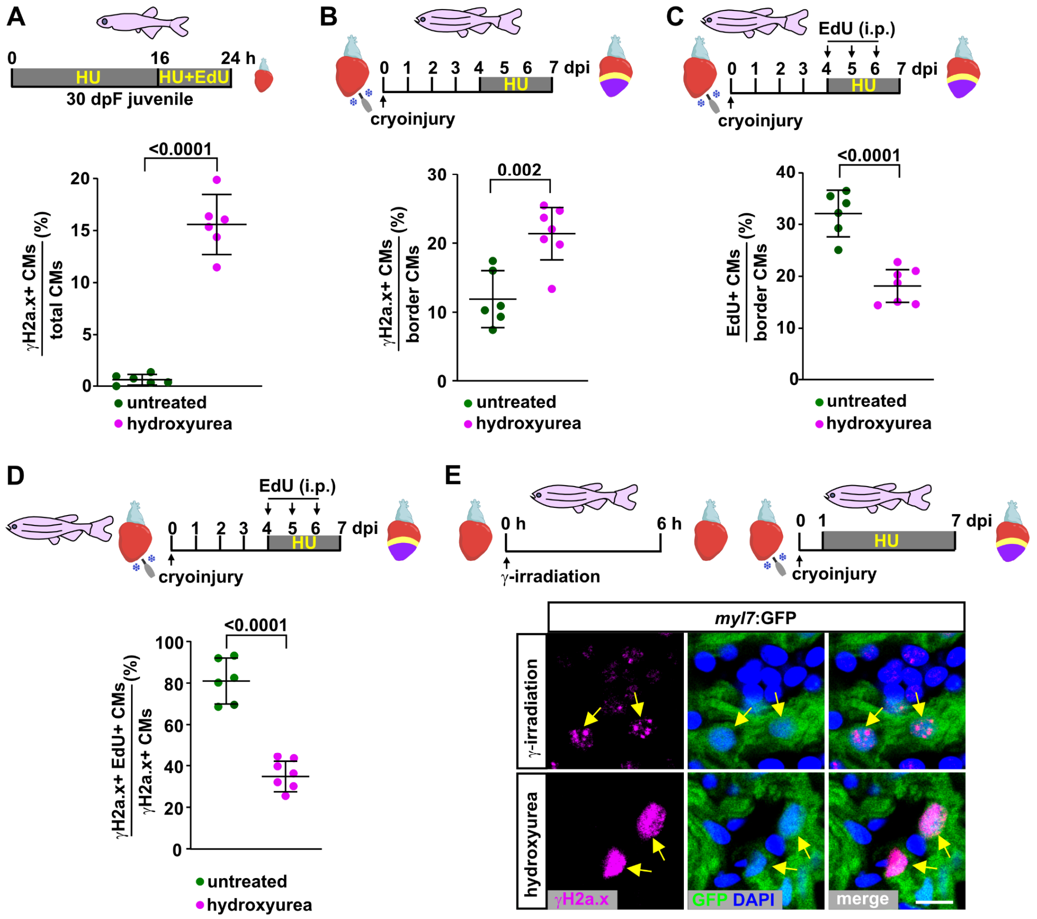

Supplementary Figure 2. *γH2a.x accumulation serves as reliable readout for replication stress in zebrafish cardiomyocytes*

(**A**) γH2a.x is hardly detectable in CMs of juvenile, uninjured fish, but induced by hydroxyurea treatment. Data are derived from the same fish as used for Figure 2B, which were also incubated with EdU as shown in the experimental scheme. $n_E = 1$, $n_A = 6$ per condition, $n_C = 4560$ untreated, 3660 HU.

(**B**) Hydroxyurea treatment in adult injured fish further enhances the fraction of γH2a.x+ CMs within 150 µm of the wound border at 7 dpi. $n_E = 1$, $n_A = 6$ untreated, 7 hydroxyurea, $n_C = 8360$ untreated, 7170 hydroxyurea.

(**C**) Hydroxyurea treatment in adult injured fish decreases the fraction of cycling wound border CMs labeled by daily i.p. injection of EdU from 4 to 6 dpi. $n_E = 1$, $n_A = 6$ untreated, 7 hydroxyurea, $n_C = 8360$ untreated, 7170 hydroxyurea.

(**D**) Hydroxyurea treatment in adult injured fish decreases the fraction of γH2a.x+ wound border CMs at 7 dpi that are also EdU+ following EdU injection from 4 to 6 dpi. $n_E = 1$, $n_A = 6$ untreated, 7 hydroxyurea, $n_C = 8360$ untreated, 7170 hydroxyurea.

(**E**) Immunofluorescence on cryosections of uninjured *myl7*:GFP transgenic hearts reveals γH2a.x accumulation in subnuclear foci in CMs 6 h after γ-irradiation (arrows). Intense pan-nuclear accumulation of γH2a.x is observed in the regenerating CMs of the wound border area at 7 dpi when fish are treated with hydroxyurea from 1 to 7 dpi (arrows). Representative image, not quantified. Scale bar, 10 µm.

(**A-D**) Data are presented as mean values. Error bars, CI 95%. Two tailed Student´s t-test. Source data are provided in the Source Data file. Some elements were created in Biorender. (Agreement number: AH27UPOG7H; Posadas, D. (2025) https://BioRender.com/w88l548)

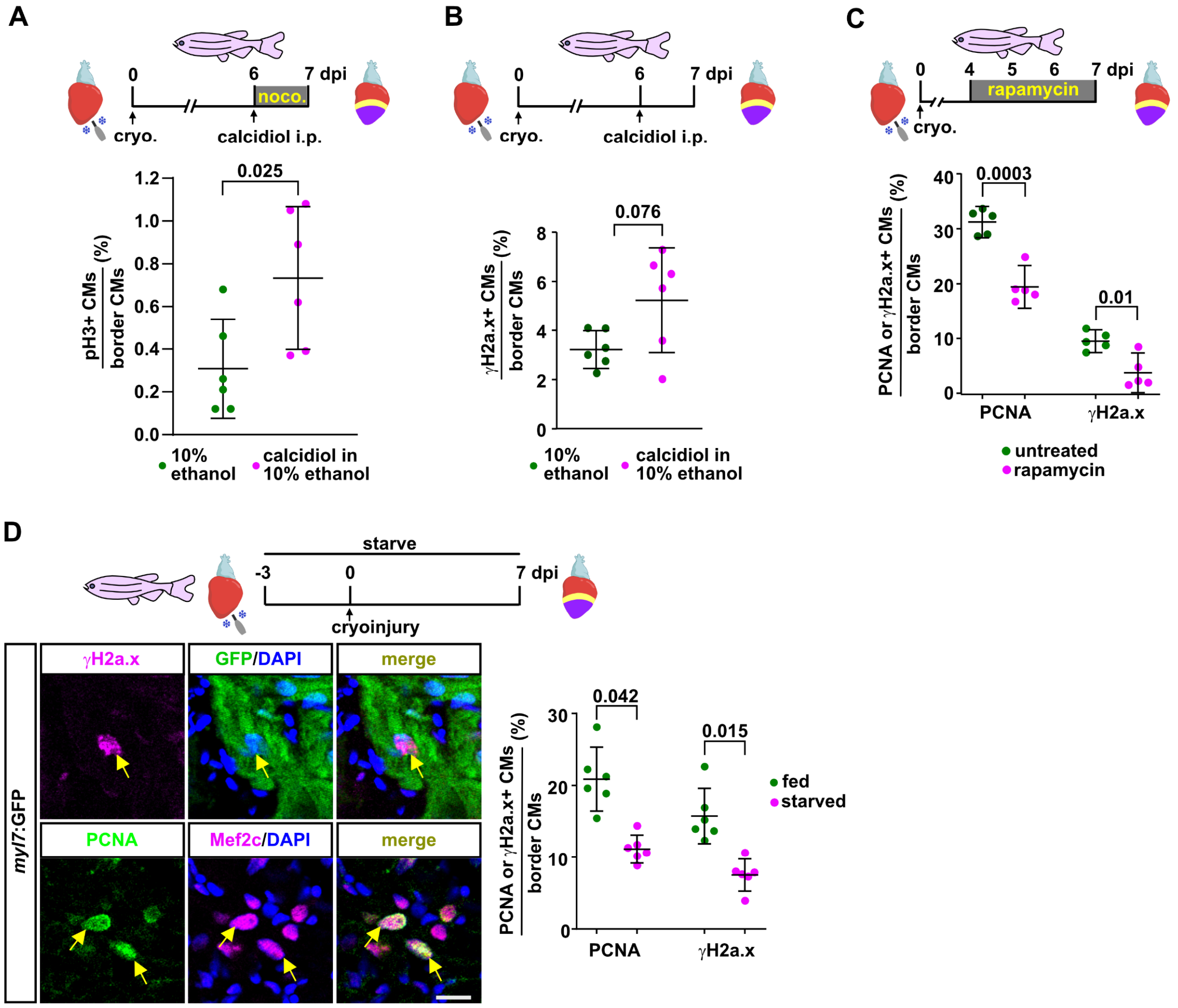

Supplementary Figure 3. *Cardiomyocyte proliferation correlates with γH2a.x accumulation*

(A) Immunofluorescence on cryosections of *myl7*:H2b-GFP transgenic hearts reveals an increase in pH3+ mitotic CMs (identified by nuclear GFP) in the wound border area 24 h after a single intra-peritoneal (i.p.) injection of Vitamin D (α-calcidiol) at 6 dpi. Note that nocodazole treatment was used to block cytokinesis, which increases the number of detectable pH3+ cells. n_E = 1, n_A = 6 for 10% ethanol, 6 for calcidiol, n_C = 6055 for 10% ethanol, 6830 calcidiol. Scale bar, 10 μm.

(B) Fish injected with α-calcidiol at 6 dpi display a strong trend towards increased fraction of γH2a.x+ wound border CMs at 7 dpi. n_E = 1, n_A = 6 per treatment, n_C = 5340 EtOH, 4890 calcidiol.

(C) Incubation of cryoinjured fish with the mTOR inhibitor rapamycin for 3 days reduces the fraction of PCNA+ and of γH2a.x+ wound border CMs at 7 dpi. n_E = 1, n_A = 5 per treatment, n_C = 10540 untreated, 9700 rapamycin.

(**D**) Immunofluorescence on cryosections of *myl7*:GFP transgenic hearts reveals PCNA and γH2a.x accumulation in wound border CMs (identified by GFP or Mef2c staining) at 7 dpi in fish that are regularly fed. When fish are starved, the fraction of PCNA+ CMs as well as the fraction of γH2a.x+ CMs decreases. n_E = 1, n_A = 6 per condition, n_C = 12270 fed, 12720 starved. Scale bar, 10 μm.

(**A-D**) Data are presented as mean values. Error bars, CI 95%. Two tailed Student´s t-test. Source data are provided in the Source Data file. Some elements were created in Biorender. (Agreement number: AH27UPOG7H; Posadas, D. (2025) https://BioRender.com/w88l548)

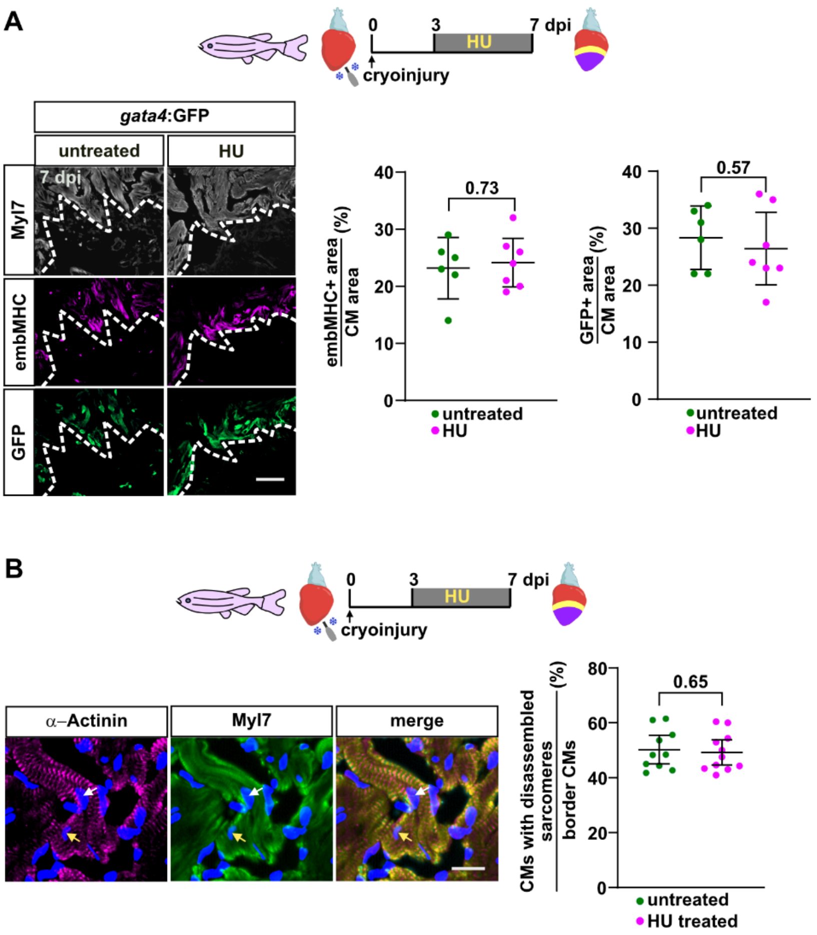

Supplementary Figure 4. *Replication stress does not affect CM dedifferentiation*

(**A**) Immunofluorescence on cryosections of *gata4*:GFP transgenic hearts reveals embryonic myosin heavy chain (embMHC) and GFP expression in Myl7+ CMs at the wound border at 7 dpi. The plots show the ventricular areas covered by anti-embMHC or anti-GFP staining relative to the 150 µm wound border zone area occupied by Myl7. Replication stress induced by hydroxyurea treatment from 3 dpi until 7 dpi does not alter these readouts of CM dedifferentiation. n_E = 2, n_A = 6 untreated, 7 HU. For embMHC the observed relative difference between the untreated and HU treated groups is 7%, the calculated smallest significant difference is 31%. For GFP the observed relative difference is 9%, the calculated smallest significant difference is 33%. These calculated smallest significant differences are smaller than the effect sizes that we have observed previously after inhibition of Wnt signaling (172% and 87% respectively) (Bertozzi, et al., 2022). We conclude that this experiment had enough power to detect biologically relevant effects. Since it did not, we conclude that HU treatment does not affect CM dedifferentiation.

(**B**) Immunofluorescence on cryosections of wild-type hearts shows that HU treatment does not alter CM dedifferentiation as determined by the disassembly of sarcomeres. The fraction of Myl7+ CMs at the wound border containing organized α-actinin+ sarcomeres at 7 dpi is plotted. White arrows show normal sarcomeres and yellow arrows show disorganized sarcomeres. n_E = 2, n_A = 10 untreated, 11 HU. n_C = 4830 untreated, 5820 HU border CMs. The observed relative difference between the untreated and HU treated groups is 7.3%, the calculated smallest significant difference 15%. These calculated smallest significant differences are smaller than the effect sizes that we have observed previously with rapamycin treated hearts (25%). We conclude that this experiment had enough power to detect biologically relevant effects. Since it did not, we conclude that HU treatment does not affect CM dedifferentiation.

(**A-B**) Data are presented as mean values. Error bars, CI 95%. Two tailed Student´s t-test. Source data are provided in the Source Data file. Some elements were created in Biorender. (Agreement number: AH27UPOG7H; Posadas, D. (2025) https://BioRender.com/w88l548)

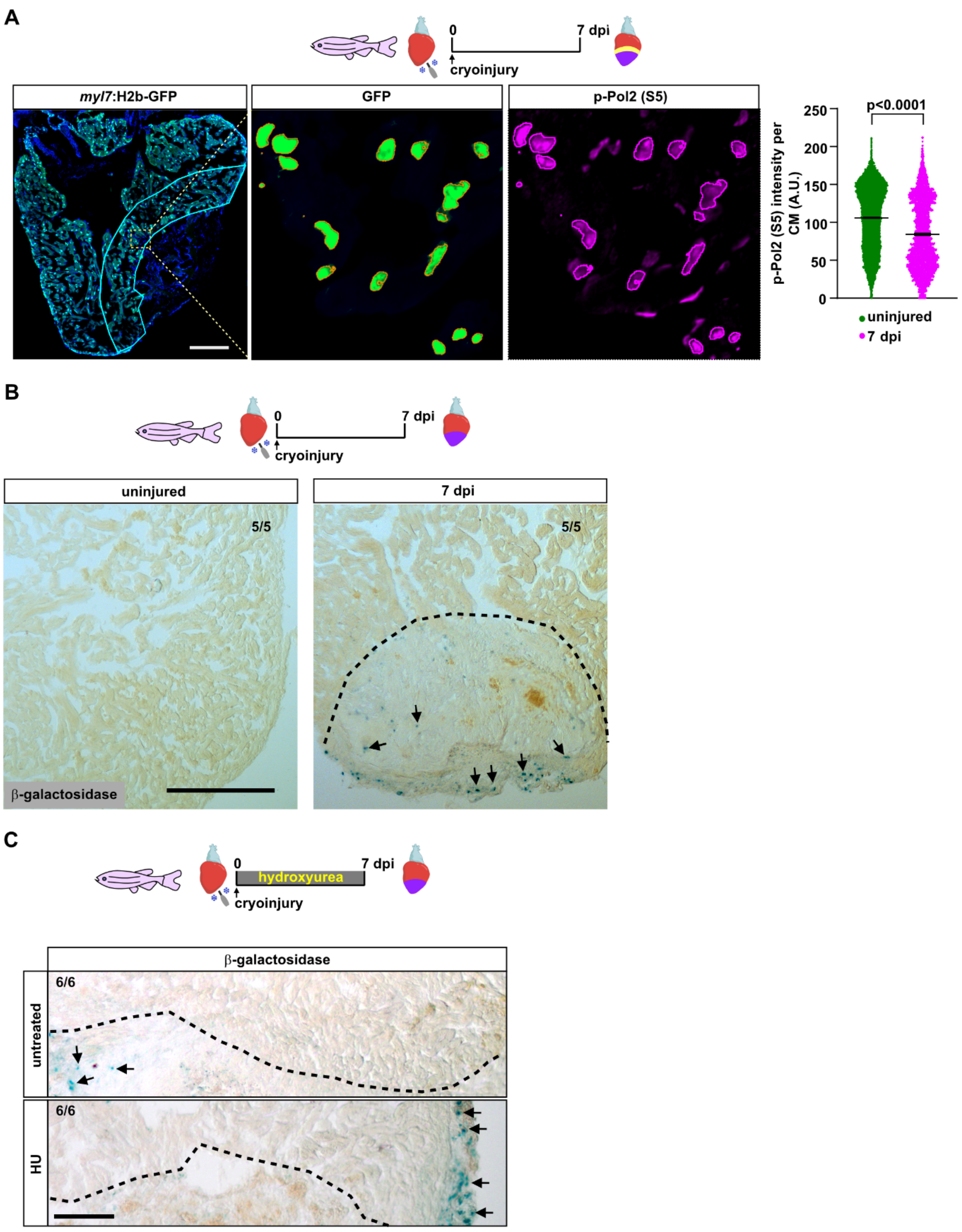

Supplementary Figure 5. *Replication stress is likely not caused by conflicts with transcription and does not induce CM senescence*

(**A**) Wound border CMs display lower overall rates of transcription than remote CMs. Immunofluorescence on cryosections of *myl7*:H2b-GFP transgenic hearts reveals levels of p-Pol2 (S5), the active, elongating phosphorylated form of RNA Polymerase II, in CMs at the wound border at 7 dpi. Selections used to segment GFP+ nuclei are indicated in yellow, dotted box indicates magnified region. The intensity of p-Pol2 (S5) staining in nuclei is plotted in the wound border region of injured hearts at 7 dpi, and in a similarly sized

myocardial region in uninjured hearts. Data are presented as mean value. Error bars, CI 95%; Two tailed Student´s t-test. $n_E = 1$, $n_A = 5$ per condition, $n_{Sections} = 8$, $n_C = 18500$ uninjured, 4000 border CMs.

(**B)** Cryosections of uninjured and regenerating hearts at 7 dpi that were stained for beta-galactosidase activity in the whole mount. At 7 dpi, sparse β-galactosidase-positive senescent cells are located within the wound and at higher density in the outermost layers of the wound, which most likely represent the epicardium that has already covered the wound (black arrows). No senescent CMs can be detected in the wound border. Images are representative of 5 hearts per group. Scale bar, 250 µm. Dashed line indicates wound border.

(C) Treatment with HU for 7 days does not induce senescence of CMs at 7 dpi. Black arrows show β-galactosidase-positive senescent cells located in the epicardial layer and wound area of treated hearts. Images are representative of 6 hearts per group. Dashed line indicates wound border. Scale bar, 100 µm.

(**A-C**) Some elements were created in Biorender. (Agreement number: AH27UPOG7H; Posadas, D. (2025) https://BioRender.com/w88l548

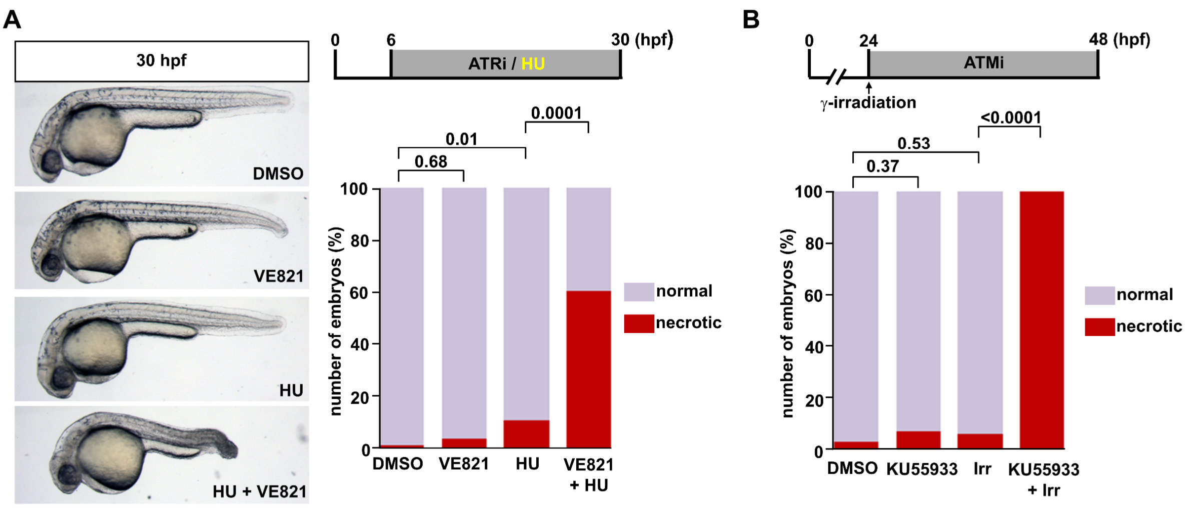

Supplementary Figure 6. *Specificity of ATM and ATR inhibitors in zebrafish*

(**A**) The ATR inhibitor VE821 is not toxic to zebrafish embryos, but causes necrosis when applied in combination with hydroxyurea. Representative images of embryos at 30 hpf (hours post fertilization) show necrosis (darkened and deformed tissue) in the tail in HU + VE821 treated embryos. Plot shows the fraction of necrotic embryos. Fisher exact test. $n_E = 1$, $n_A = 30$ per treatment.

(**B**) The ATM inhibitor KU55933 is not toxic to zebrafish embryos, but causes necrosis when combined with γ-irradiation. Fisher exact test. $n_E = 1$, $n_A = 100$ per treatment.

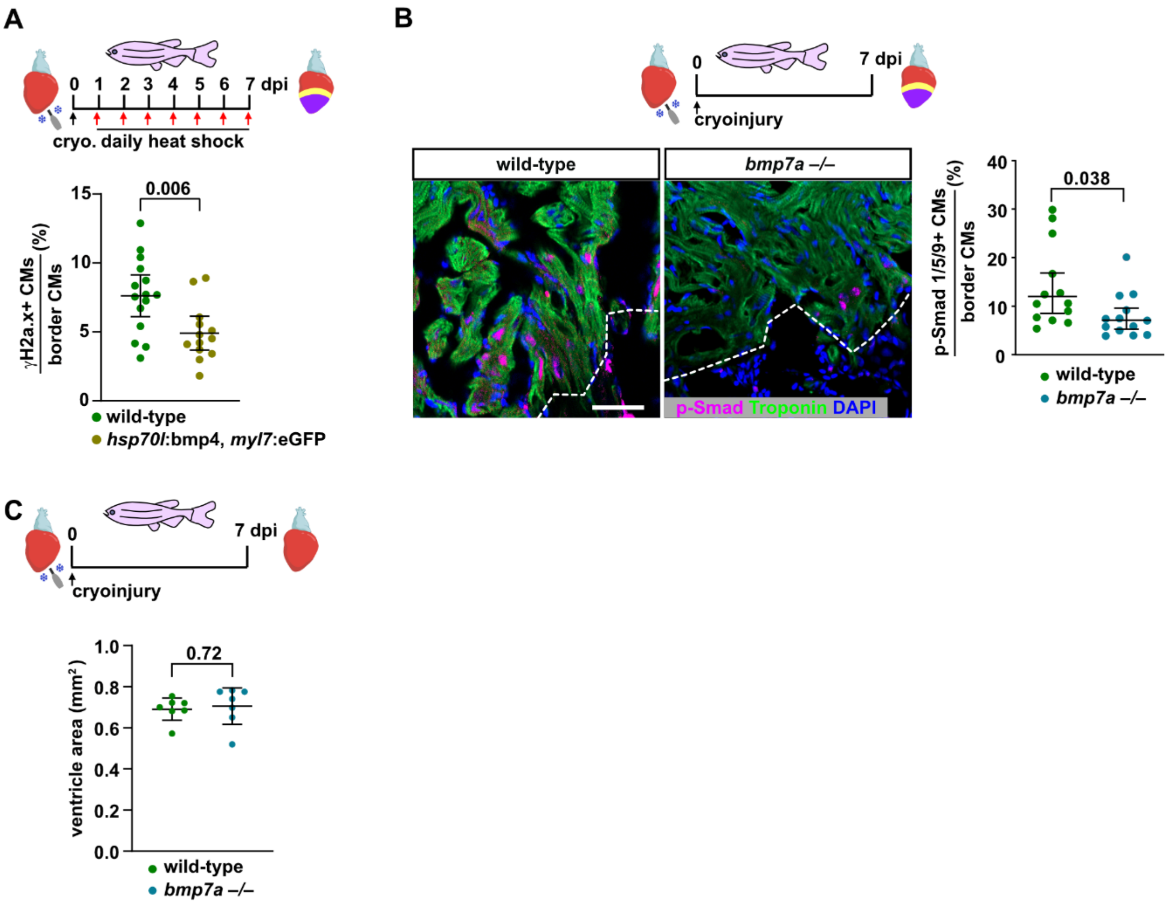

Supplementary Figure 7. *Additional data on bmp4 overexpression and bmp7a mutant hearts*

(**A**) Bmp-GOF using *hsp70l*:bmp4 transgenic fish results in a decrease in the fraction of γH2a.x+ CMs in the wound border at 7 dpi compared to heat-shocked wild-type sibling hearts. n_E = 2, n_A = 15 wild-type, 13 *hsp70l*:bmp4, n_C = 7923 wild-type, 7850 *hsp70l*:bmp4.

(**B**) *bmp7a –/–* mutant fish show reduced p-Smad1/5/9 in wound border CMs compared to wild type siblings at 7 dpi. n_E = 2, n_A = 12 wild-type, 13 *bmp7a –/–*. n_C = 4600 wild-type, 6200 *bmp7a –/–*. Scale bar, 100 µm.

(**C**) Ventricles of *bmp7a –/–* mutant fish are similar in size to their wild-type siblings indicating that the hearts do not display obvious developmental defects. Data points represent total ventricle area derived from individual hearts at 7 dpi. n_E = 1, n_A = 7 wild-type, 7 *bmp7a –/–*, $n_{Sections}$ = 8-12 per heart. The observed relative difference between wild-type and mutant groups is 2%, the calculated smallest significant difference 13%.

(**A-C**) Data are presented as mean values. Error bars, CI 95%. Two tailed Student´s t-test. Source data are provided in the Source Data file. Some elements were created in Biorender. (Agreement number: AH27UPOG7H; Posadas, D. (2025) https://BioRender.com/w88l548)

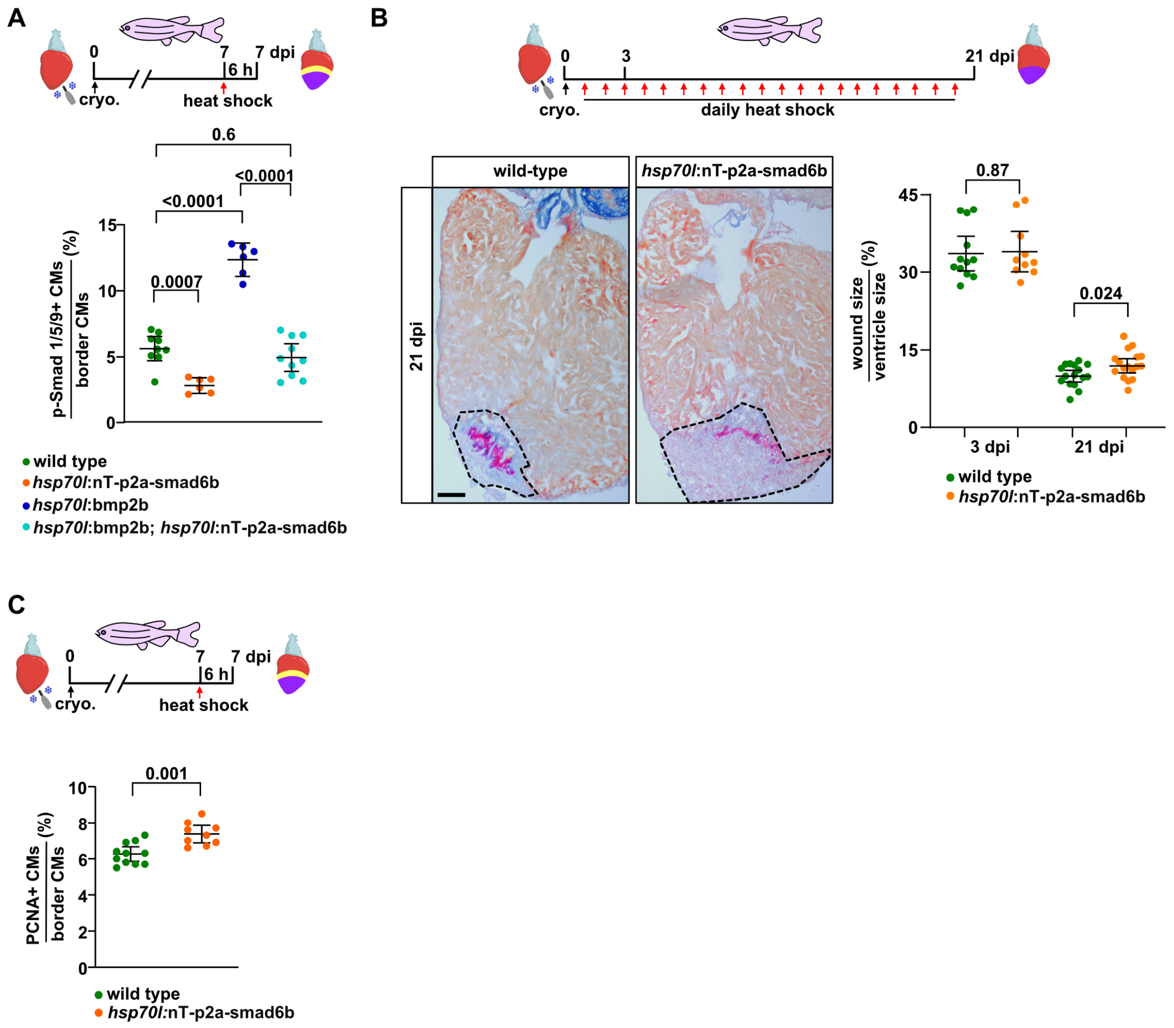

Supplementary Figure 8. *Smad6b and bmp2b overexpression cancel each other's ability to alter BMP signaling, smad6b overexpression increases the fraction of PCNA+ CMs and reduces scar resolution.*

(**A**) Bmp-LOF using *hsp70l*:nT-p2a-smad6b reduces the fraction of p-Smad1/5/9+ wound border CMs 6 h after a single heat shock at 7 dpi, while *hsp70l*:bmp2b mediated Bmp-GOF increases it. Yet, in *hsp70l*:nT-p2a-smad6b; *hsp70l*:bmp2b double transgenics the fraction of p-Smad1/5/9+ CMs is comparable to that in heat-shocked wild-types, indicating that under these conditions the ability of the two transgenes to modulate Smad signaling cancel each other. n_E =2, n_A = 9 wild-type, 6 *hsp70l:*nT-p2a-smad6b, 6 *hsp70l*:bmp2b, 10 *hsp70l:*nT-p2a-smad6b *x hsp70l*:bmp2b, n_C = 12000 total CMs across all groups.

(B) AFOG staining on sections of cryoinjured *hsp70l*:nT-p2a-smad6b transgenics and wild-type siblings reveals slightly larger wound sizes in transgenics (black dashed lines) at 21 dpi following daily heat-shock. Note that no differences are detected at 3 dpi, indicating that the additional wounding conditions were similar in both groups. Myocardium, brown; collagen, blue; fibrin, red. n_E =2, n_A = 16 wt, 17 *hsp70l:*nT-p2a-smad6b. Scale bar, 100 µm.

(**C**) In *hsp70l*:nT-p2a-smad6b transgenic hearts the fraction of PCNA+ CMs at the wound border is increased 6 h after a single heat-shock at 7 dpi. n_E =2, n_A = 11 wild-type, 9 *hsp70l:*nT-p2a-smad6b, n_C = 6800 wild-type, 6950 *hsp70l:*nT-p2a-smad6b.

(**A-B**) Ordinary one-way ANOVA with Bonferroni correction. (**C**) Two tailed Student´s t-test. (**A-C**) Data are presented as mean values. Error bars, CI 95%. Source data are provided in the Source Data file. Some elements were created in Biorender. (Agreement number: AH27UPOG7H; Posadas, D. (2025) https://BioRender.com/w88l548)

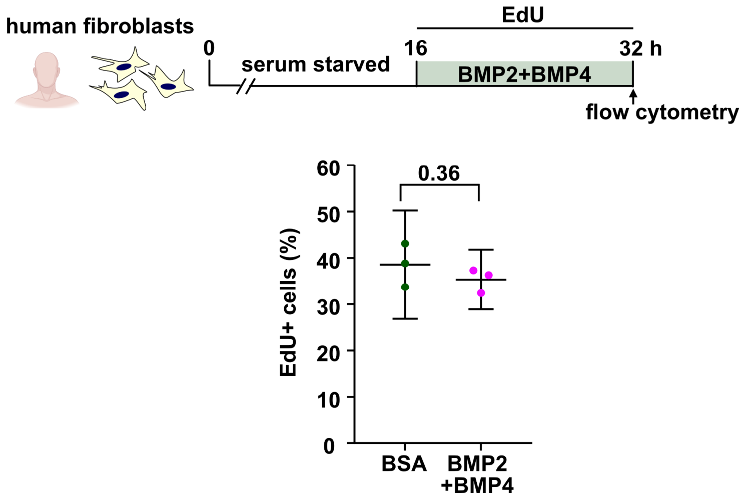

Supplementary Figure 9. *EdU incorporation in human fibroblasts*

EdU incorporation determined by flow cytometry in human primary fibroblasts shows no increase in replication upon BMP treatment. Data points represent the percentage of EdU incorporated cells. Data are presented as mean value. Error bars, 95% CI; Two tailed Student´s t-test, n_E =3, n_{cells}= 5000 per replicate. Some elements were created in Biorender. (Agreement number: CH27UPPBB6; Posadas, D. (2025) https://BioRender.com/n30v908)

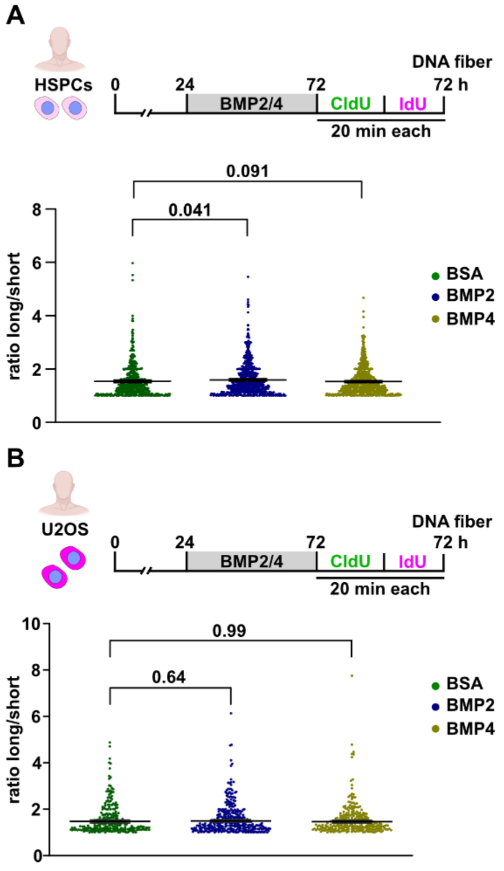

Supplementary Figure 10. *Pretreatment with BMP ligands does not affect replication fork stalling*

(A) Long vs short track ratios measured from DNA fibers containing both CldU and IdU tracks in Figure 8A indicate a minor, yet significant increase in fork asymmetry in hematopoietic stem and progenitor cells treated with BMP2, but not with BMP4 ligands. Data points represent the ratio of long to short tracks within individual CldU + IdU labeled fibers. n_E =3, n_{fibers} = 600 per treatment.

(B) Long vs short track ratios measured from DNA fibers containing both CldU and IdU tracks in Figure 8B shows no change in fork asymmetry in U2OS cells treated with BMP2 or BMP4 ligands. Data points represent the ratio of long to short tracks within individual CldU + IdU labeled fibers. n_E =2, n_{fibers} = 250 per treatment.

(A-B) Data are presented as mean values. Error bars, CI 95%. Kruskal-Wallis followed by Dunn´s correction. Source data are provided in the Source Data file. Some elements were created in Biorender (Agreement number: CH27UPPBB6; Posadas, D. (2025) https://BioRender.com/n30v908)

Supplementary References

Bertozzi, A. *et al.* Is zebrafish heart regeneration "complete"? Lineage-restricted cardiomyocytes proliferate to pre-injury numbers but some fail to differentiate in fibrotic hearts. *Dev Biol* **471**, 106-118, doi:10.1016/j.ydbio.2020.12.004 (2021).

Supplementary Tables

Supplementary Table 1. Drugs, chemicals, ligands

Name	Manufacturer	Identifier
Alfacalcidiol (vitamin D agonist)	Selleckchem	Cat# S1468
Rapamycin	Selleckchem	Cat# S1039
Hydroxyurea	Merck	Cat# H8627
VE-821 (ATR Kinase Inhibitor)	Selleckchem	Cat# S8007
KU55933 (ATM Kinase Inhibitor)	Selleckchem	Cat# S1092
Nocodazole	Merck	Cat# M1404
ClickTech EdU Cell Proliferation Kit 647 for IM	Baseclick	Cat# EdU647IM100+IV-S
Senescence β-Galactosidase Staining	Cell Signaling	Cat# 9860S
KASP Master mix	LGC Biosearch Technology	Cat# KBS-1050-101
KASP Assay primer mix	LGC Biosearch Technology	Cat# KBS-2100-100
4′,6-Diamidin-2-phenylindol -dihydrochlorid (DAPI)	Merck	Cat# 32670
DMSO	Merck	Cat# D2650
Recombinant Human BMP-2	RandD systems	Cat# 355-BM-010
Recombinant Human BMP-4	RandD systems	Cat# 314-BP-010
Recombinant Human BMP-7	RandD systems	Cat# 354-BP-010
SB202190 (p38 kinase inhibitor)	Sigma	Cat# S7067
PD0332991 (cdk4/6 inhibitor)	Selleckchem	Cat# S1579
Bouin´s solution	Sigma	Cat# HT10132-1L
Phosphomolybdic acid	Sigma	Cat# HT153
Aniline blue	Sigma	Cat# 415049
Orange G	Fluka	Cat# 73580
Acid fuchsin	Sigma	Cat# F8129
Human Methylcellulose Complete Media	R&D Systems	Cat# HSC003
Nuclease P1	NEB	Cat# M0660S
Alkaline Phosphatase	Sigma	Cat# P5931
Competitive DNA Damage ELISA Kit	Thermo Fisher Scientific	Cat# EIADNAD

Supplementary Table 2. Primers for RT-qPCR on zebrafish samples

Gene	Identifier (ZFIN)	Sense primer	Antisense primer
Rad51	ZDB-GENE-040426-2286	GTCATCACTAACCAGGTTGTAGC	ATCTCCCACTCCATCAGCATTAAT
Rad54l	ZDB-GENE-040426-968	ATAGAGGAGAAGATCCTCCAGAGAC	AGTTGGAGAGATCACAGGTACAGTC
Xrcc5	ZDB-GENE-041008-108	AGGAGCACTGAGTATTACACCAAGA	AATGACAGGAACTGATTTGCTTCT
Gtf2h4	ZDB-GENE-030131-6779	ACACCCAGTAATGCTTAAACAGACC	AGAATCTCTTAACCTCGCTGTGTC
Fen1	ZDB-GENE-031112-11	TAATTCAGTTCATGTGTGCTGAGAA	CGTAGCACTTGTCTTTTGTTTCTTT
Rpa2	ZDB-GENE-010131-3	GTGCTAACATGATGCTAGTCAATGG	GATCTTCGTCGATGGTGGAGAAAAT
Chaf1a	ZDB-GENE-030131-5366	CACAAACTCTTCTACCACACCCTC	CAAGGATGTGTTGATGTCCTGTTC
Atm	ZDB-GENE-040809-1	AACAATGGAAGTTATGAGGAGTTCT	CACGCTCCGCCACTTTATTGAA
Atr	ZDB-GENE-070912-458	TGTGAGGTCATACTAAGACTCATGA	TATTCCTGTTCTTGATCACTCCCTG
Prkdc	ZDB-GENE-030131-9008	CTTGCTGCTCAACACTATGGATG	CCCTTATACTCCGGCATCTTCTC
18s rRNA	ZDB-RRNAG-180607-2	CGCTATTGGAGCTGGAATTACC	GAAACGGCTACCACATCCAA
Ubb	ZDB-GENE-050411-10	CTCAGATTAGAACCGACAGTCTTAGG	GCAACACAACATGAATAAAATAATGGGAAA
Eef1a1l1	ZDB-GENE-990415-52	TTCCTCTTTCTGTTACCTGGCAA	CTTCTCGATGGTTCTCTTGTCGATT

Supplementary Table 3. Primary antibodies

Short name	Full name	Source	Identifier	Application
p-Smad1/5/9	Rabbit monoclonal Phospho-Smad1 (Ser463/465)/ Smad5 (Ser463/465)/ Smad9 (Ser465/467)	Cell Signaling Technology	Cat# 13820 RRID:AB_2493181	Immunostaining: Zebrafish cryosections
PCNA	Mouse monoclonal Proliferating Cell Nuclear Antigen	Dako	Cat# M0879 RRID:AB_2160651	Immunostaining: Zebrafish cryosections
Mf20	Mouse monoclonal MF20 (which detects sarcomeric Myosin heavy chain – MHC)	Developmental Studies Hybridoma Bank	Cat# MF20 RRID:AB_2147781	Immunostaining: Zebrafish cryosections
Myl7	Rabbit polyclonal Myl7	GeneTex	Cat# GTX128346, RRID:AB_2885759	Immunostaining: Zebrafish cryosections
Caspase-3	Rabbit monoclonal Caspase 3	BD Biosciences	Cat# 559565 RRID:AB_397274	Immunostaining: Zebrafish cryosections
GFP	Chicken polyclonal GFP	Abcam	Cat# Ab13970 RRID:AB_2936447	Immunostaining: Zebrafish cryosections
embMHC	Mouse monoclonal MYH7	DSHB	Cat# N2.261 RRID:AB_531790	Immunostaining: Zebrafish cryosections
α-actinin (sarcomeric)	Mouse monoclonal	Sigma	Cat# A7732 RRID:AB_2221571	Immunostaining: Zebrafish cryosections
pH3	Mouse monoclonal Phospho-Histone 3 (Ser10) 6G3	Cell Signaling	Cat# 9706S RRID:AB_331748	Immunostaining: Zebrafish cryosections
p-Rpa32 (s33)	Rabbit polyclonal RPA32 Phospho (S33)	Bethyl Laboratories	Cat# A300-246A	Immunoblotting: Zebrafish ventricles
Gapdh	Rabbit monoclonal GAPDH	Cell Signaling	Cat# 2118S RRID:AB_561053	Immunoblotting: Zebrafish ventricles
Mef2	Rabbit polyclonal Mef2c	Santa Cruz Biotechnology	Cat# SC313 (discontinued)	Immunostaining: Zebrafish cryosections
Troponin	Cardiac troponin (CT3)	DSHB	Cat# CT3	Immunostaining:Zebrafish cryosections
Troponin	Anti-cardiac troponin I	Abcam	Cat#ab47003	Immunostaining: Primary neonatal mouse cardiomyocyte
γH2a.x	Rabbit polyclonal histone H2A.XS139ph (phospho Ser139)	Genetex	Cat# GTX127342 RRID:AB_2885642	Immunostaining and immunoblotting: zebrafish cryosections and zebrafish ventricles
γH2a.x	Anti-phospho-Histone H2A.X (Ser139) Antibody, clone JBW301	Millipore	Cat# 05-636	Immunostaining: Primary human neonatal dermal fibroblasts
γH2a.x	Phospho-Histone H2A.X (Ser139) (20E3)	Cell Signaling	Cat# 9718	Immunostaining: Primary neonatal mouse cardiomyocytes, U2OS cell line
p-Pol2 (S5)	Anti-RNA polymerase II CTD repeat YSPTSPS (phospho S5) antibody	Abcam	Cat# ab5131	Immunostaining: Zebrafish heart cryosections
BrdU	Anti-BrdU detecting IdU (mouse)	BD Biosciences	Cat# 347580	DNA fiber spreading assays
BrdU	Anti-BrdU detecting CldU (rat, monoclonal, clone BU1/75 (ICR1))	BioRad	Cat# OBT0030	DNA fiber spreading assays

Supplementary Table 4. Secondary antibodies

Name	Source	Identifier	Application
Goat anti-Rabbit IgG (H+L) Highly Cross-Adsorbed Secondary Antibody, Alexa Fluor™ 488	Invitrogen	Cat# A11034 RRID:AB_2576217	Immunostaining
Goat anti-Rabbit IgG (H+L) Highly Cross-Adsorbed Secondary Antibody, Alexa Fluor™ 555	Invitrogen	Cat# A21429 RRID:AB_2535850	Immunostaining
Goat anti-Mouse IgG (H+L) Highly Cross-Adsorbed Secondary Antibody, Alexa Fluor™ 633	Invitrogen	Cat# A21052 RRID:AB_2535719	Immunostaining
Goat anti-Mouse IgG (H+L) Highly Cross-Adsorbed Secondary Antibody, Alexa Fluor 555	Invitrogen	Cat# A21424 RRID:AB_141780	Immunostaining
Goat anti-Rabbit IgG (H+L) Cross-Adsorbed Secondary Antibody, Alexa Fluor™ 633	Invitrogen	Cat# A21070 RRID:AB_2535731	Immunostaining
IRDye® 680RD Goat anti-Rabbit IgG Secondary Antibody	Licor	Cat# 926-68071	Immunoblotting

.
