# Supplementary material for: BMP signaling promotes zebrafish heart regeneration via alleviation of replication stress
Source: Nat Commun. 2025 Feb 17;16:1708. doi: 10.1038/s41467-025-56993-6 (PMC11832743; doi:10.1038/s41467-025-56993-6)
Supplement: Supplementary file 3 — Description of Additional Supplementary Files [file 41467_2025_56993_MOESM3_ESM.pdf]

Description of Additional Supplementary Files

File Name: Supplementary Data 1
Description: Contains gene expression data of different stages of zebrafish heart regeneration in whole ventricles after ventricular resection.

File Name: Supplementary Data 2
Description: Lists genes representing the GO term “DNA repair” that were found to be enriched at 7 dpi in regenerating vs. sham injured hearts.
