# Supplementary material for: BMP signaling promotes zebrafish heart regeneration via alleviation of replication stress
Source: Nat Commun. 2025 Feb 17;16:1708. doi: 10.1038/s41467-025-56993-6 (PMC11832743; doi:10.1038/s41467-025-56993-6)
Supplement: Supplementary file 6 — Reporting Summary [file 41467_2025_56993_MOESM6_ESM.pdf]

nature portfolio

Corresponding author(s): Gilbert Weidinger

Last updated by author(s): 2025-01-31

Reporting Summary

Nature Portfolio wishes to improve the reproducibility of the work that we publish. This form provides structure for consistency and transparency in reporting. For further information on Nature Portfolio policies, see our Editorial Policies and the Editorial Policy Checklist.

Please do not complete any field with "not applicable" or n/a. Refer to the help text for what text to use if an item is not relevant to your study.
For final submission: please carefully check your responses for accuracy; you will not be able to make changes later.

Statistics

For all statistical analyses, confirm that the following items are present in the figure legend, table legend, main text, or Methods section.

n/a	Confirmed	
☐	☒	The exact sample size (n) for each experimental group/condition, given as a discrete number and unit of measurement
☒	☐	A statement on whether measurements were taken from distinct samples or whether the same sample was measured repeatedly
☐	☒	The statistical test(s) used AND whether they are one- or two-sided<br>*Only common tests should be described solely by name; describe more complex techniques in the Methods section.*
☒	☐	A description of all covariates tested
☐	☒	A description of any assumptions or corrections, such as tests of normality and adjustment for multiple comparisons
☐	☒	A full description of the statistical parameters including central tendency (e.g. means) or other basic estimates (e.g. regression coefficient) AND variation (e.g. standard deviation) or associated estimates of uncertainty (e.g. confidence intervals)
☐	☒	For null hypothesis testing, the test statistic (e.g. F, t, r) with confidence intervals, effect sizes, degrees of freedom and P value noted<br>*Give P values as exact values whenever suitable.*
☒	☐	For Bayesian analysis, information on the choice of priors and Markov chain Monte Carlo settings
☒	☐	For hierarchical and complex designs, identification of the appropriate level for tests and full reporting of outcomes
☒	☐	Estimates of effect sizes (e.g. Cohen's d, Pearson's r), indicating how they were calculated

Our web collection on statistics for biologists contains articles on many of the points above.

Software and code

Policy information about availability of computer code

Data collection	Confocal microscopy was performed using the Leica SP8 confocal microscope using the LAS software. Image analysis was performed using ImageJ software. Graphs were plotted and statistical significance calculated using a combination of Grpahpad Prism V9 and Microsoft Excel. Custom made microarray for zebrafish probes was designed using an Agilent platform. Microarray analysis was performed using Limma package from Bioconductor.
Data analysis	Microarray was analysed using Limma package from Bioconductor. GO enrichment analysis was performed using R package clusterProfiler. For Gene Ontology, enrichment score plots were generated using R package enrichplot. Differential gene expression analysis of single cell RNA seq data was performed using Scanpy 1.10.

For manuscripts utilizing custom algorithms or software that are central to the research but not yet described in published literature, software must be made available to editors and reviewers. We strongly encourage code deposition in a community repository (e.g. GitHub). See the Nature Portfolio guidelines for submitting code & software for further information.

Data

Policy information about availability of data

All manuscripts must include a data availability statement. This statement should provide the following information, where applicable:

- Accession codes, unique identifiers, or web links for publicly available datasets
- A description of any restrictions on data availability
- For clinical datasets or third party data, please ensure that the statement adheres to our policy

scRNA seq data from cardiomyocytes is available under GEO accession number GSE130940. Microarray transcriptomics data is available as Supplementary Files.

Research involving human participants, their data, or biological material

Policy information about studies with human participants or human data. See also policy information about sex, gender (identity/presentation), and sexual orientation and race, ethnicity and racism.

Reporting on sex and gender	While human samples have been used based on voluntary donations of cord blood or bone marrow hematopoietic stem and progenitor cells, the study does not systematically consider sex/gender of donors.
Reporting on race, ethnicity, or other socially relevant groupings	Not applicable
Population characteristics	Not applicable
Recruitment	Not applicable
Ethics oversight	Not applicable

Note that full information on the approval of the study protocol must also be provided in the manuscript.

Field-specific reporting

Please select the one below that is the best fit for your research. If you are not sure, read the appropriate sections before making your selection.

☒ Life sciences ☐ Behavioural & social sciences ☐ Ecological, evolutionary & environmental sciences

Life sciences study design

All studies must disclose on these points even when the disclosure is negative.

Sample size	When possible experiments were performed with a minimum sample size of 6 per group. Care was taken to minimize animal use yet achieve \`n´numbers to achieve statistical power.
Data exclusions	For most experiments, a data point was excluded only in cases of technical failures (like staining inadequacy). For HPSC colony formation assays, data was excluded only when hydroxyurea induced suppression lay outside of 20-80%.
Replication	Replication of each experiment wherever possible has been performed and has been mentioned in the figure legends.
Randomization	When wildtypes were used, animals were assigned to control and test groups by the investigator without any systematic randomization. For experiments relating to the hsp70l:nT-p2a-smad6b, genotyping was performed after experimental intervention during image acquisition thus achieving full randomization. For other transgenics and mutants due to limitations with genotyping protocol, no randomization was performed.
Blinding	For most experiments investigators were blind to the data sets mainly during data analysis. For experiments relating to the hsp70l:nT-p2a-smad6b, investigators were blind during the entire course of the experiment till image quantification. For other transgenics, investigators knew the genotype of the animals during experiments. However, care was taken to blind the data during image quantification and data was matched to genotype after quantification. For experiments related to cell culture, blinding was not always possible.

Reporting for specific materials, systems and methods

We require information from authors about some types of materials, experimental systems and methods used in many studies. Here, indicate whether each material, system or method listed is relevant to your study. If you are not sure if a list item applies to your research, read the appropriate section before selecting a response.

Materials & experimental systems

n/a	Involved in the study
	☒ Antibodies
	☒ Eukaryotic cell lines
	☐ Palaeontology and archaeology
	☒ Animals and other organisms
	☐ Clinical data
	☐ Dual use research of concern
	☐ Plants

Methods

n/a	Involved in the study
	☐ ChIP-seq
	☐ Flow cytometry
	☐ MRI-based neuroimaging

Antibodies

Antibodies used	Manuscript contains Table 3 in the Supplementary section with details of all primary and secondary antibodies and their usage.
Validation	All antibodies used are commercially available. gamma-H2ax antibodies were validated by ensuring that they detect increased signals after hydroxyurea treatment or gamma-irradiation of samples. Most other antibodies are routinely used in the lab and have been validated previously.

Eukaryotic cell lines

Policy information about cell lines and Sex and Gender in Research

Cell line source(s)	Human U2OS cell line and Human neonatal foreskin fibroblasts procured from ATCC repository.
Authentication	No authentication was performed for cell lines
Mycoplasma contamination	Verified to be absent
Commonly misidentified lines (See ICLAC register)	Not applicable

Palaeontology and Archaeology

Specimen provenance	*Provide provenance information for specimens and describe permits that were obtained for the work (including the name of the*
Specimen deposition	*Indicate where the specimens have been deposited to permit free access by other researchers.*
Dating methods	*If new dates are provided, describe how they were obtained (e.g. collection, storage, sample pretreatment and measurement), where*

☐ Tick this box to confirm that the raw and calibrated dates are available in the paper or in Supplementary Information.

Ethics oversight	*Identify the organization(s) that approved or provided guidance on the study protocol, OR state that no ethical approval or guidance*

Note that full information on the approval of the study protocol must also be provided in the manuscript.

Animals and other research organisms

Policy information about studies involving animals; ARRIVE guidelines recommended for reporting animal research, and Sex and Gender in Research

Laboratory animals	Zebrafish: The following previously published transgenic or mutant fish lines were used: hsp70l:nog3fr14tg ; hsp70l:bmp2bfr13tg; myl7:GFPtwu34Tg ; 14.8gata4:GFPae1; myl7:H2b-GFPzf521Tg; hsp70l:bmp7b,myl7:eGFPaf5Tg ; hsp70l:bmp4; myl7:eGFPaf1Tg ; bmp7aty68a/ty68a mutant.<br>Mouse: wild-type C57BL/6 mouse
Wild animals	No wild animals were used in this study.
Reporting on sex	Fish of both sexes have been used when available in all experiments. Data were analyzsed separately for both sexes, but no significant differences were found. Thus, we report combined date in the manuscript.
Field-collected samples	Not applicable
Ethics oversight	Ethical committee details are provided in manuscript. All experiments involving zebrafish and mouse were approved by the state of

Note that full information on the approval of the study protocol must also be provided in the manuscript.

Clinical data

Policy information about clinical studies

All manuscripts should comply with the ICMJE guidelines for publication of clinical research and a completed CONSORT checklist must be included with all submissions.

Clinical trial registration	Not applicable
Study protocol	Not applicable
Data collection	Not applicable
Outcomes	Not applicable

Dual use research of concern

Policy information about dual use research of concern

Hazards

Could the accidental, deliberate or reckless misuse of agents or technologies generated in the work, or the application of information presented in the manuscript, pose a threat to:

No	Yes	
☒	☐	Public health
☒	☐	National security
☒	☐	Crops and/or livestock
☒	☐	Ecosystems
☒	☐	Any other significant area

Experiments of concern

Does the work involve any of these experiments of concern:

No	Yes	
☒	☐	Demonstrate how to render a vaccine ineffective
☒	☐	Confer resistance to therapeutically useful antibiotics or antiviral agents
☒	☐	Enhance the virulence of a pathogen or render a nonpathogen virulent
☒	☐	Increase transmissibility of a pathogen
☒	☐	Alter the host range of a pathogen
☒	☐	Enable evasion of diagnostic/detection modalities
☒	☐	Enable the weaponization of a biological agent or toxin
☒	☐	Any other potentially harmful combination of experiments and agents

Plants

Seed stocks	Not applicable
Novel plant genotypes	Not applicable
Authentication	Not applicable

ChIP-seq

Data deposition

☐ Confirm that both raw and final processed data have been deposited in a public database such as GEO.

☐ Confirm that you have deposited or provided access to graph files (e.g. BED files) for the called peaks.

Data access links<br>*May remain private before publication.*	Not applicable
Files in database submission	Not applicable
Genome browser session<br>(e.g. UCSC)	Not applicable

Methodology

Replicates	Not applicable
Sequencing depth	Not applicable
Antibodies	Not applicable
Peak calling parameters	Not applicable
Data quality	Not applicable
Software	Not applicable

Flow Cytometry

Plots

Confirm that:

☐ The axis labels state the marker and fluorochrome used (e.g. CD4-FITC).

☐ The axis scales are clearly visible. Include numbers along axes only for bottom left plot of group (a 'group' is an analysis of identical markers).

☐ All plots are contour plots with outliers or pseudocolor plots.

☐ A numerical value for number of cells or percentage (with statistics) is provided.

Methodology

Sample preparation	not applicable
Instrument	*Identify the instrument used for data collection, specifying make and model number.*
Software	*Describe the software used to collect and analyze the flow cytometry data. For custom code that has been deposited into a* [illegible]
Cell population abundance	*Describe the abundance of the relevant cell populations within post-sort fractions, providing details on the purity of the* [illegible]
Gating strategy	*Describe the gating strategy used for all relevant experiments, specifying the preliminary FSC/SSC gates of the starting cell* [illegible]

☐ Tick this box to confirm that a figure exemplifying the gating strategy is provided in the Supplementary Information.

Magnetic resonance imaging

Experimental design

Design type	*Indicate task or resting state; event-related or block design.*
Design specifications	*Specify the number of blocks, trials or experimental units per session and/or subject, and specify the length of each trial* [illegible]
Behavioral performance measures	*State number and/or type of variables recorded (e.g. correct button press, response time) and what statistics were used* [illegible]

Acquisition

Imaging type(s)	*Specify: functional, structural, diffusion, perfusion.*

Field strength | *Specify in Tesla*

Sequence & imaging parameters | *Specify the pulse sequence type (gradient echo, spin echo, etc.), imaging type (EPI, spiral, etc.), field of view, matrix size,*

Area of acquisition | *State whether a whole brain scan was used OR define the area of acquisition, describing how the region was determined.*

Diffusion MRI ☐ Used ☐ Not used

Preprocessing

Preprocessing software | *Provide detail on software version and revision number and on specific parameters (model/functions, brain extraction,*

Normalization | *If data were normalized/standardized, describe the approach(es): specify linear or non-linear and define image types used for*

Normalization template | *Describe the template used for normalization/transformation, specifying subject space or group standardized space (e.g.*

Noise and artifact removal | *Describe your procedure(s) for artifact and structured noise removal, specifying motion parameters, tissue signals and*

Volume censoring | *Define your software and/or method and criteria for volume censoring, and state the extent of such censoring.*

Statistical modeling & inference

Model type and settings | *Specify type (mass univariate, multivariate, RSA, predictive, etc.) and describe essential details of the model at the first and*

Effect(s) tested | *Define precise effect in terms of the task or stimulus conditions instead of psychological concepts and indicate whether ANOVA*

Specify type of analysis: ☐ Whole brain ☐ ROI-based ☐ Both

Statistic type for inference | *Specify voxel-wise or cluster-wise and report all relevant parameters for cluster-wise methods.*

(See Eklund et al. 2016)

Correction | *Describe the type of correction and how it is obtained for multiple comparisons (e.g. FWE, FDR, permutation or Monte Carlo).*

Models & analysis

n/a	Involved in the study
☐	☐ Functional and/or effective connectivity
☐	☐ Graph analysis
☐	☐ Multivariate modeling or predictive analysis

Functional and/or effective connectivity | *Report the measures of dependence used and the model details (e.g. Pearson correlation, partial correlation,*

Graph analysis | *Report the dependent variable and connectivity measure, specifying weighted graph or binarized graph,*

Multivariate modeling and predictive analysis | *Specify independent variables, features extraction and dimension reduction, model, training and evaluation*

This checklist template is licensed under a Creative Commons Attribution 4.0 International License, which permits use, sharing, adaptation, distribution and reproduction in any medium or format, as long as you give appropriate credit to the original author(s) and the source, provide a link to the Creative Commons license, and indicate if changes were made. The images or other third party material in this article are included in the article's Creative Commons license, unless indicated otherwise in a credit line to the material. If material is not included in the article's Creative Commons license and your intended use is not permitted by statutory regulation or exceeds the permitted use, you will need to obtain permission directly from the copyright holder. To view a copy of this license, visit http://creativecommons.org/licenses/by/4.0/
